# NIRS Hemodynamic Response to Methylphenidate in Children with Attention Aeficit Hyperactivity Disorder: First Administration, Titration Phase and Associations with Clinical Severity

**DOI:** 10.1192/j.eurpsy.2022.181

**Published:** 2022-09-01

**Authors:** A. Crippa, S. Grazioli, E. Rosi, M. Mauri, F. Villa, E. Maggioni, V. Diwadkar, P. Brambilla, M. Pozzi, M. Molteni, M. Nobile

**Affiliations:** 1 Scientific Institute IRCCS ’E. Medea’, Child Psychopathology Unit, Bosisio Parini, Italy; 2 Fondazione IRCCS Ca’ Granda Ospedale Maggiore Policlinico, Department Of Neurosciences And Mental Health, Milan, Italy; 3 Wayne State University School of Medicine, Departments Of Psychiatry And Behavioral Neurosciences, Detroit, United States of America; 4 Scientific Institute IRCCS ’E. Medea’, Clinical And Translational Pharmacology, Bosisio Parini, Italy

**Keywords:** methylphenidate, adhd, Near Infrared Spectroscopy (NIRS)

## Abstract

**Introduction:**

Attention deficit hyperactivity disorder (ADHD) is a neurodevelopmental disorder characterized by lack of self-regulation and deficits in organizing behaviors in response to emotional stimuli. Methylphenidate (MPH) is one of the most effective psychostimulant drugs for ADHD, however, a possible predictive utility of brain hemodynamic data related to MPH administration and its relation to clinical symptomatology is still not clear. To address these questions, we used Near Infrared Spectroscopy (NIRS) technology, a non-invasive optical technique that allows to investigate the effect of psychopharmacological treatment on cortical hemodynamics.

**Methods:**

Twenty children with ADHD underwent a three-waves study and 25 healthy controls were recruited at W1. At W2 children with ADHD received first MPH administration and at W3 they reached the titration phase. At each phase children performed - during NIRS recording - an emotional continuous performance task with visual stimuli of different emotional content. Clinical data were also collected at W1 and W3. We investigated the relationship among the difference between NIRS activation at W2 and W1 (Delta1) and W3 and W2 (Delta2), for each subject, task condition and brain region. Lastly, we investigated correlations between the Delta1 and clinical symptomatology indexes at W1 and between Delta2 and clinical data at W3.

**Conclusions:**

Our study results suggest that hemodynamic changes in right prefrontal region probably induced by first MPH administration could predict hemodynamic changes related to MPH titration phase. These biological indexes could be associated to clinical evidences related not only to core ADHD symptoms but also to affective correlates.

**Disclosure:**

No significant relationships.

